# The Impact of Transcutaneous Electrical Nerve Stimulation (TENS) on the Consecutive Stages of Labour and Perinatal Outcomes—A Retrospective Cohort Study

**DOI:** 10.3390/jcm14103445

**Published:** 2025-05-15

**Authors:** Agata Michalska, Anna Blazuk-Fortak, Aleksandra Gladys-Jakubczyk, Daniel Wolder, Grzegorz Swiercz

**Affiliations:** 1 Collegium Medicum, Jan Kochanowski University, 25-317 Kielce, Poland; michalska.agata@ujk.edu.pl (A.M.); aleksandra.gladys-jakubczyk@ujk.edu.pl (A.G.-J.); d.wolder@wp.pl (D.W.); grzegorzswiercz60@gmail.com (G.S.); 2 Department of Obstetrics and Gynecology, Regional Hospital in Kielce, 25-736 Kielce, Poland

**Keywords:** episiotomy, labour, neonate, perineal tearing, transcutaneous electrical nerve stimulation

## Abstract

**Background**: Over recent years, several pain management techniques have been proposed to control labour pain, including pharmacological and non-pharmacological interventions. Transcutaneous electrical nerve stimulation (TENS) is considered a safe, non-invasive, easily applicable, and inexpensive pain relief method. This study aimed to investigate the impact of TENS on consecutive labour stages and on maternal and neonatal outcomes. **Methods**: This retrospective, single-centre cohort study covered a two-year period (1 January 2022–31 December 2023). A total of 1451 women met the inclusion criteria. TENS was applied in 203 of them. In 54.67% of cases, TENS was combined with water immersion and, in 42.85%, with water immersion and Entonox (N_2_O and O_2_ mixture). Two groups of patients that either made use of TENS, or not, to reduce labour pain, were compared to assess the effect of TENS on the course of labour and the condition of the newborn. **Results**: The women in the TENS group experienced a significantly longer first stage of labour. There was no statistically significant difference between the groups in terms of oxytocin usage, perineal tearing, episiotomy, and umbilical cord blood pH. The simultaneous application of TENS and water immersion contributed to prolonging the first stage of labour relative to their independent effects. **Conclusions**: The application of TENS may prolong the first stage of labour, without increasing the rate of perineal tearing and episiotomy and without any adverse effects on the condition of the newborn.

## 1. Introduction

Childbirth is associated with pain, which is described by women as intense, demanding, and difficult to bear. It is characterized by a gradual escalation, cyclical occurrence of contractions, and high intensity, increasing with greater cervical dilatation. This pain is mediated by sympathetic fibres to the ganglia of the posterior nerve roots located at the T10-L1 spinal levels and by the pudendal nerve to the ganglia of the posterior nerve roots located at spinal levels S2–S4. The intensity of pain sensations associated with childbirth is individually variable. The perception of pain may depend on obstetric history, previous experience with pain, notion of pregnancy and labour, emotional and physical status, extent of fear and anxiety during the actual labour, and sense of control over one’s body and the birthing process [1,2,3,4].

Pain management in labour comprises pharmacological interventions along with non-pharmacological ones (e.g., water immersion, acupressure, massage, reflexotherapy, transcutaneous electrical nerve stimulation—TENS, interferential electrical stimulation, warm and cold packs, aromatherapy, relaxation, hypnosis, biofeedback, intracutaneous or subcutaneous sterile water injection, fully immersive virtual reality solutions). Apart from the therapeutic approach of an attending medical professional, other essential components are also required, i.e., effective communication, comfortable and relaxing environment, counselling, and specific educational input for patients and families. In everyday clinical practice, these methods are frequently combined [1,2,5,6].

Transcutaneous electrical nerve stimulation (TENS) is a non-pharmacological method of pain relief during labour. The term TENS refers to a group of low-frequency impulse electrical currents used for pain relief (neuromodulation, anaesthetic). In clinical terms, TENS might be applied at varying frequencies, intensities, and pulse durations of stimulation. The frequency of stimulation is broadly classified as high-frequency (>50 Hz or 100 Hz and above, better tolerated), low-frequency (2–10 Hz), or burst TENS (bursts of high-frequency stimulation applied at a much lower frequency). The analgesic effect of TENS and the time of analgesia depend on the frequency of the stimulus and variable moments of endorphin release. With the use of low frequencies (2–10 Hz), it takes longer to achieve pain relief, but the effect lasts longer. High-frequency stimulation (90–150 Hz) causes a more rapid but less sustainable effect due to the serotonin neurotransmission and metabolism. It is also possible to use a burst frequency, in which the high-frequency doses are interspersed with two or three low-frequency doses. This is to overcome the effect of habituation to the same long-term frequency usage [7]. The current is supplied from one or more circuits and electrode patches, which are placed on the skin at the level of the sacrum and the border of the thoracic and lumbar spinal segments or at select acupuncture points. The electrode patches may be placed on specific acupoints (transcutaneous electrical acupoint stimulation—TEAS) [8]. TENS during labour may be combined with other pain relief methods, although it may not be applied during water immersion. This method of analgesia is considered safe, non-invasive, easily applicable, and inexpensive.

It may well be applied by a midwife, or a nurse, in consultation with the pregnant woman. Pregnant women may operate the TENS unit on their own by way of regulating the intensity of the impulses during contractions, which can help create a sense of control over the birthing process. TENS is widely used for pain relief, even though there is limited evidence that TENS actually reduces pain during labour [9,10,11,12].

There is no significant evidence of TENS’s side effects on both the mother and the newborn. However, before TENS implementation, certain precautions should be taken. It is important to check whether the patient has implanted medical devices that could interfere with current [7]. TENS equipment can have an impact on CTG monitoring, so when the clinical situation concerning foetal heart rate monitoring is not clear, this fact should be taken into consideration [12].

Previous studies have mainly focused on the pain relief as the primary outcome. The present study, though, aimed to determine the effect of TENS on the course of labour, with particular consideration of its duration and the occurrence of perineal trauma, as well as the effect on the birth status of the newborn.

## 2. Materials and Methods

This single-centre, retrospective, observational study had 1451 women enrolled. A retrospective analysis of data on the childbirth and condition of the newborn, extracted from electronic hospital records, was carried out. No informed consent was required as all the data were anonymized. All cases of singleton, cephalic, vaginal delivery in the classic lithotomy position, with no epidural analgesia, were selected for analysis.

During the study period, no significant changes were encountered in the protocols pertaining to the interventions in the delivery room. Among the non-pharmacological methods of pain relief during labour, water immersion, massage, TENS, warm and cold packs, aromatherapy, positioning, breathing exercises, and Entonox (inhalable analgesic gaseous mixture of nitrous oxide and oxygen in a 1:1 ratio) were applied. Only the water immersion (at least 30 min in the bath or in the shower, up to 2–3 times during the labour; the exact time of the procedure was not given) and TENS were recorded in the documentation. These two methods were never used simultaneously. During the first stage of labour, TENS was applied to the lower back (electrode placements on T10-L1 and S2–S4) at a frequency of 100 Hz, with an individually adjustable intensity. The patient also decided when to stop the stimulation. These electrostimulation parameters and electrode patch positionings are the most commonly used, as evidenced in research [9,10]. The duration of electrical stimulation was not reported in the medical records. The duration of the first stage of labour is calculated from the onset of regular contractions until full cervical dilation (10 cm) is reached.

Data were collected on maternal and obstetric characteristics: age, pregnancy, parity, delivery variables. Newborn-related characteristics such as birth weight (grams) and umbilical cord venous blood pH were also collected. The main outcome assessments were the duration of labour and the incidence of perineal trauma. The main neonatal outcome analysis measure was the pH of the umbilical cord venous blood.

Statistical analysis was performed with the aid of the “R studio v2022” software package using the “R” programming language. To assess the effect of TENS on the study endpoints, a logistic regression model was used for dichotomous endpoints and a general linear model for endpoints that were quantitative variables (time of the first and second stage of labour, umbilical cord blood pH). Multivariate modelling was carried out when significant results were obtained in the univariate models, using backward elimination in the selection of variables and assessment of potential interactions.

**Figure 1:**
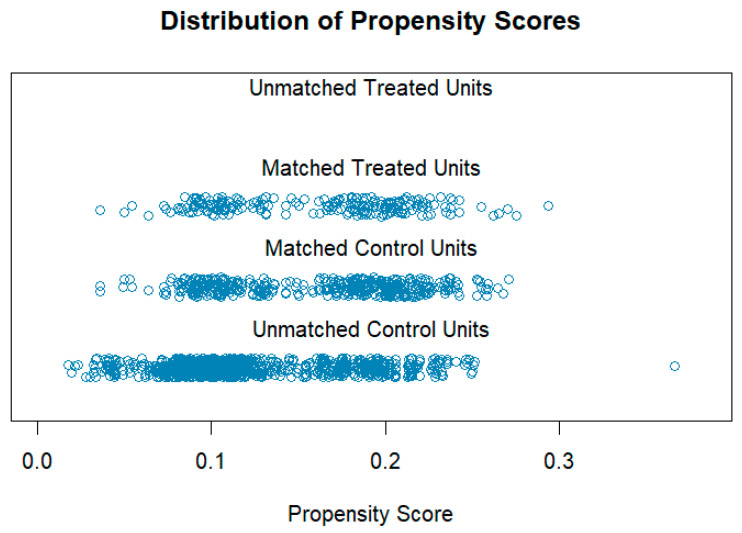
Propensity score matching—visualization of the case selection and group matching (treated—TENS group, control—non-TENS group).

In this study, women were allocated into the TENS group (203) and the non-TENS group (1248). Subsequently, the non-TENS group were separated in a 2:1 ratio to the number of women in the TENS group in terms of the similarity of maternal, obstetric, and neonatal variables (maternal age, gestational age, gravidity, parity, vaginal birth after caesarean—VBAC, newborn’s body weight). Propensity score matching to distinguish the group of 406 non-TENS women was also used (Figure 1).

This study was granted endorsement by the Bioethics Committee of the Jan Kochanowski University of Kielce.

## 3. Results

The group under study consisted of 609 women (203 in the TENS group, 406 in the non-TENS group). The median age of the mothers was 30 years, and the median gestational age was 39 weeks. Most of the women (61.08%) were primiparous. Most childbirths took place during the day (spanning 7.00 a.m.–11.59 p.m.). Induced deliveries predominated in the study group (63.55%). In 111/203 (54.67%) cases, TENS was combined with water immersion and, in 87/203 (42.85%) of them, additionally with Entonox (Table 1).

There were no statistically significant differences between the TENS and the non-TENS groups in terms of maternal age, gravidity, parity, gestation age, type of delivery, time of delivery (at night or during the day), and newborn’s weight (Table 2). It was noted that TENS was applied less frequently in women with a preterm rupture of membranes (PROM). Entonox (*p* < 0.001) and water immersion (*p* = 0.027) were more frequently applied in the TENS group. The first stage of labour in the TENS group lasted significantly longer (*p* = 0.036). In the spontaneous deliveries, the first stage of labour continued for 261.92 min (109.58 SD) and, in the induced ones, for 239.18 min (130.90 SD) in the TENS group vs. 221.43 min (101.50 SD) and 222.60 min (113.75 SD) in the non-TENS group. There was no statistically significant difference in the duration of the second stage of labour, although it was shorter in the non-TENS group for both spontaneous and induced deliveries.

There were no statistically significant differences between the groups in terms of oxytocin administration, perineal tearing, episiotomy, and umbilical cord blood pH. The percentage of newborns with pH < 7.2 was higher in the group of parturients who did not receive any TENS intervention. No differences in the rates of perineal trauma were noted between the groups. The rate of intact perineums was similar (21.67% non-TENS group vs. 21.18% TENS group). The rate of perineal tearing was higher in the TENS group (non-significant difference).

**Figure 2:**
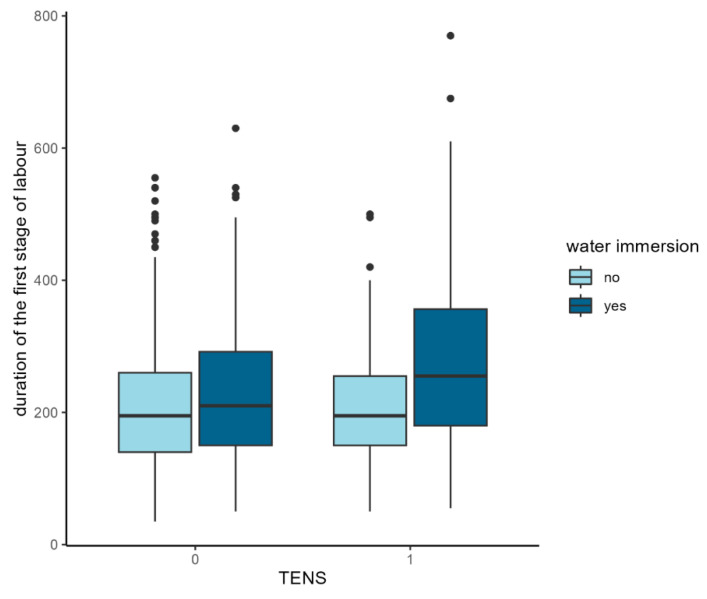
TENS, water immersion, and duration of the first stage of labour data association.

Regression models were used to describe the effect of the variables on the duration of the first and the second stage of labour, the presence of perineal trauma (episiotomy and perineal lacerations), and the umbilical cord blood pH. The application of TENS significantly affected the duration of the first stage of labour only (*p* = 0.022), contributing to its extension by 22.62 min. A significant interaction was observed between water immersion and TENS (*p* = 0.007). The application of both methods simultaneously contributed to a prolongation of the first stage of labour relative to their independent effects (Figure 2).

Based on the univariate models, a multivariate model describing the effect of the selected variables for the first stage of labour was made. A significant effect was observed for primiparity (*p* < 0.001) and for the water immersion–TENS interaction (*p* = 0.007) (Table 3). The R^2^ value was 0.1809; the model accounted for 18.09% of the variability. After excluding primiparity from the model, the effect of the water immersion–TENS interaction remained statistically significant. The R2 value was 0.037; the model accounted for 3.7% of the variability.

## 4. Discussion

The pain associated with childbirth is often described as the most intense pain experience of a lifetime. The physiological response of the parturient’s body to labour pain significantly impacts the condition of the foetus and the progress of labour itself [13,14]. Pain management in labour comprises both pharmacological and non-pharmacological interventions [1,2,5]. There is a wide range of options available, although the pharmacological methods of pain relief are much preferred. Non-pharmacological interventions are perceived as a form of helping women cope with pain during childbirth rather than as a pain-relieving therapy. They are often combined to optimize the analgesic effect and create a satisfying delivery experience. Among the most popular pain relief methods listed by the late pregnancy survey, nitrous oxide and oxygen mixture (Entonox) (79%), bath (63%), massage (44%), epidural analgesia (37%), breathing techniques (28%), mental training (19%), TENS (17%), and acupuncture (14%) were referenced [15]. Evidence from the studies investigating the effect of non-pharmacological pain relief methods (including TENS) on the course of labour and maternal outcomes is often disparate and, hence, difficult to assess. In this study, we did not observe any adverse effects of TENS on maternal and newborn health. The long-term effects on maternal health may be caused by operative procedures during the course of delivery. The use of TENS does not seem to influence the rate of caesarean sections or other obstetric interventions during labour; therefore, it does not affect the process of recuperation after giving birth. The data on neonatal outcomes are limited [12]. Available studies show that neonatal Apgar scores were not dependent on TENS application during labour [16]. Thus, the benefits and risks of TENS for labour pain relief and methodology of intervention are still to be conclusively established [10,11].

In the group under study, TENS was applied in 14% of women. The rate of TENS application was similar to the one reported in the Swedish study (17%) [15] but lower than in the Finnish study (31%) [17]. TENS, as a non-pharmacological method of pain relief, does not seem to have any serious adverse effects on the women’s or newborns’ condition. The findings of the present study on the overall safety of TENS application are similar to those already referenced in previous studies [1,10,12,13,14,16,17,18]. No statistically significant differences were found between the study groups in terms of umbilical cord blood pH, oxytocin administration, episiotomy, and perineal tearing.

The analgesic effect of TENS is facilitated through peripheral, spinal, and supraspinal mechanisms. High-frequency TENS can alter the excitability of peripheral nociceptors to reduce the afferent input to the central nervous system. The spinal effect works via the “pain-gate” mechanism, by interrupting the pain impulses with sensory impulses (gate-control theory) and decreasing inflammation-induced dorsal horn neuron sensitization. There are different opioids released with high- or low-frequency TENS stimulation (endorphins, methionine enkephalin, and dynorphin A) [9,19,20,21]. Beta-endorphins are endogenous opioids that give adaptive responses to stress and pain during labour. Excessive maternal stress in labour may lead to excessive (supraphysiologic) beta-endorphins, which in turn may inhibit oxytocin and slow the labour itself. Optimal levels of beta-endorphins promoting the progress of labour vary among women [22]. TENS produces analgesia by activating endogenous inhibitory mechanisms in the central nervous system, involving opioid, GABA, serotonin, muscarinic, and cannabinoid receptors [20].

It has been proposed that, by reducing attendant stress and pain, the actual length of labour may also be cut down. Dowswell et al. [12] concluded that TENS did not seem to have an effect on the length of labour, likewise Thuvarakan et al. [10]. Shahoei et al. [23] noted a reduction in length in the first stage of labour in the group of women making use of TENS (TENS frequency data not provided). No statistically significant difference in the mean duration of the second and third stages of labour was reported, though. In the Brazilian study, a sequence of interventions, i.e., walking around, alternation of postures, TENS (frequency 80 Hz), and a warm bath aided the labour. The parturients who received this multimodal intervention had a shorter active phase of labour [18].

On the other hand, Samadzadeh et al. [24] report a prolongation of the first stage of the labour after the TENS intervention (frequency 50 Hz). In an Egyptian study, the first stage of labour was longer in the parturients making use of TENS (15–70 HZ) as compared with those on pharmacotherapy, although the difference remained statistically insignificant. Our own study indicated that the first and second stage of labour were significantly shorter in the group of parturients with no TENS application. The variation in the effect of TENS may be connected to the release of oxytocin caused by somatosensory stimulation. It is well known that oxytocin stimulates the contractile function of the uterus that induces labour. On the other hand, high levels of plasma oxytocin reduce blood pressure and anxiety sensation [25], which may set off a feeling of calmness and relaxation and consecutively prolong labour, especially in the first stage. Transcutaneous stimulation is most commonly used simultaneously with other analgesia, and their effect on labour is conjoint. Moreover, there are other labour characteristics not connected to analgesia that impact its duration. For example, a vertical position shortens the second phase of delivery [26]. We used the rectangular pulse shape with a frequency of 100 Hz and a width of 200 μs. A Turkish study assessed the effects of TENS applied at different frequencies (100 Hz, 80–100 Hz) on the hormone levels and on the course of labour. A group of women treated with TENS at 80–100 Hz demonstrated a significant increase in oxytocin and endorphin levels and a significant decrease in post-test plasma cortisol level, labour pain perception, anxiety levels, and the duration of the successive labour stages as compared with the group treated with TENS at 100 Hz, placebo, and the control group [27]. In many similar papers, the particular frequency used was not given. However, when summing up the exact data mentioned before, the pain-relieving frequency was high, from 50 to 100 Hz. Following those conclusions, the authors suggest the use of the high-frequency, low-intensity, and short-duration electrical pulses (50–100 Hz). The intensity of the impulse should be notably over the sensational threshold (the sensation of tingling) and below the movement threshold. TENS can be used from 30 min even up to a few hours.

Research evidence indicates that specific and different analgesic mechanisms, including different opioid receptors, are activated by different frequencies of TENS [9,19,28]. According to Gibson et al. [19], a critical factor in optimizing the effectiveness of TENS, regardless of the frequency, is the actual intensity (a strong, non-painful sensation). TENS-induced analgesia should peak during or immediately after its application. Repeated treatment with the same frequency of TENS (low-frequency or high-frequency TENS) would produce tolerance to its analgesic effects [9,19,21,28].

The clinical effectiveness of TENS depends on a number of factors (stimulation parameters, e.g., frequency, intensity, and pulse duration; site of application, medications, wide variation in response). Farra et al. [16] believe that clinical and socioeconomic characteristics, including age, education, and subcutaneous adipose tissue thickness, are equally important. This may at least in part account for the discrepancy in the results of various studies assessing the effect of TENS on labour. The effect of TENS can differ within people with the same medical condition as it does not cure pathology itself but is used for symptomatic pain relief. The experience of pain is very complex and may be influenced by social (interpersonal relationships), psychological (past medical experience, especially connected to labour), and biological factors (pain sensation threshold of nociceptors) [29]. It was proven that transcutaneous stimulation depends on the thickness of skin. A thicker dermis layer and stratum corneum can decrease TENS’s analgesic effect. Moreover, the increase in frequency within the rage of 1 to 100 Hz does not intensify the reception of stimuli [30]. Prenatal education has an impact on pain experience during labour, as well. Studies show that women who attended antenatal classes assess the intrapartum pain as less intense [31]. They may also find TENS more beneficial. The feel of labour pain is modulated by a woman’s attitude to this experience. Studies show that physiological delivery is a source of empowerment for the parturient and that helps her bear with pain. Physical activity also has an impact on pain feeling. It was proven that, when the woman is in vertical positions, especially in the second phase of labour, it has an analgesic effect. When analysing the TENS’s mode of action, it raises oxytocin levels. This helps in dealing with stress and pain in the advanced first stage of delivery and causes a stronger sense of joy right after giving birth, which also influences the feel of pain [32].

In the present study, a significant interaction between the application of water immersion and TENS was observed. The application of both methods simultaneously contributed to a significant prolongation of the first stage of labour relative to their independent effects. Water immersion during labour is applied for relaxation and pain relief. This intervention stimulates the secretion of endorphins, reduces catecholamine release, and consequently alleviates overall labour discomfort [33]. According to Cluett et al. [34] and Ergin et al. [35], there is still insufficient evidence on the effect of water immersion on the actual duration of any stage of labour. Moreover, the evidence supporting the use of non-pharmacological interventions comes most often from studies in which only one method was applied. The number of studies assessing the effectiveness of the sequential or combined application of these interventions is limited. Gallo et al. [36] evaluated the effect of a series of non-pharmacological interventions (e.g., Swiss ball exercises, massage, warm shower) on maternal and neonatal outcomes. The interventions reduced the severity of pain, with no adverse effects on obstetric outcomes (condition of the perineum, obstetric complications) and neonatal outcomes. The same results were noted in the Brazilian study [18] in which the women under study made use of walking around, alternated their postures, had TENS applied, and had a warm bath. In the light of these reports, the relationship between the application of pain relief methods and the activity and mobility of the parturient should be investigated.

During this study, no oral non-opioid medications were used with TENS during the course of labour. We wanted to particularly focus on non-pharmacological pain relief methods. However, there is evidence that non-opioid medications, such as paracetamol are effective in intrapartum pain relief without causing any adverse effects on the mother and neonate, too [37]. It is also notable that the efficacy of opioid analgesia during labour does not significantly exceed acetaminophen’s effectiveness [38]. Thereupon, its use simultaneously with TENS should be considered.

### Study Limitations

This study addressed a group of women giving birth within a single maternity ward, which consequently limits the generalizability of the results. The electronic medical record system does not take into account certain type of data, i.e., the actual duration of TENS application; overall activity and mobility of the parturient during the first stage of labour; or the use of breathing techniques, massage, relaxation, and other non-pharmacological methods of pain relief.

## 5. Conclusions

Our study provides medical evidence supporting the lack of negative effects of TENS on both maternal and neonatal outcomes. The interaction between water immersion and TENS accounts for the prolongation of the first stage of labour and should, therefore, draw the attention of researchers to the cumulative effects of applying non-pharmacological pain relief methods in combination. But, first and foremost, both the actual benefits and potentially adverse effects of combining diverse pain relief strategies should be given much deeper insights in the ongoing research.

Furthermore, health care professionals should be closely familiar with the neurophysiological and hormonal mechanisms of physiological birth, as well as with the effects exerted by various agents applied in pain relief. By understanding the way TENS actually works, the intervention strategies, based on optimizing the frequency and/or intensity during the actual application, may be much better tailored to the specific needs of women at childbirth.

## Figures and Tables

**Table 1:** Maternal, obstetric, and birth characteristics of vaginal deliveries (*n* = 609).

Characteristics	Range; Mean (SD)/(*n*%)
Maternal age (years)	18–44; 30.58 (4.45)
Gestational age (weeks)	35–41; 39.04 (1.16)
Gravidity	1–6; 1.706 (0.965)
Parity	1–4; 1.517 (0.739)
Primiparous	372 (61.08)
Multiparous	237 (38.92)
GDM *	45 (7.39)
PROM **	124 (20.36)
Spontaneous delivery	222 (36.45)
Induced delivery	387 (63.55)
Vacuum extraction	20 (3.28)
VBAC ***	5 (0.82)
Oxytocin	314 (51.56)
Entonox (N_2_O + O_2_)	319 (52.38)
Water immersion	290 (47.62)
TENS	203 (33.33)
Duration 1st stage (minutes)	35–770; 230.25 (115.03)
Duration 2nd stage (minutes)	2–158; 36.22 (29.07)
Delivery at night	145 (23.81)
Delivery during the day	464 (76.19)
Episiotomy	349 (57.30)
Perineal tearing (I–IV)	129 (21.19)
Intact perineum	131 (21.51)
Birth weight (grams)	2650–4750; 3384.58 (400.47)
Umbilical cord blood pH	7.045–7.535; 7.35 (0.07)

* GDM—gestational diabetes mellitus, ** PROM—preterm rupture of membranes, *** VBAC—vaginal birth following a caesarean section.

**Table 2:** Maternal, obstetric, and birth characteristics—comparison between the TENS and the non-TENS groups.

Variable		Non-TENS (*n* = 406)	TENS (*n* = 203)	*p*	Statistical Test *
Maternal age (years)		30.55 (4.48 SD)	30.65 (4.41 SD)	0.792	T
Gestational age (weeks)		39.03 (1.15 SD)	39.08 (1.19 SD)	0.415	W
Parity		1.52 (0.75 SD)	1.51 (0.71 SD)	0.994	C
Primiparous	No	157 (38.67%)	80 (39.41%)	0.93	C
	Yes	249 (61.33%)	123 (60.59%)		
GDM	No	377 (92.86%)	187 (92.12%)	0.869	C
	Yes	29 (7.14%)	16 (7.88%)		
PROM	No	336 (82.76%)	149 (73.40%)	0.009	C
	Yes	70 (17.24%)	54 (26.60%)		
Spontaneous delivery	No	262 (65.76%)	125 (64.04%)	0.741	C
	Yes	144 (34.24%)	78 (35.96%)		
Vacuum extraction	No	392 (96.55%)	197 (97.04%)	0.936	C
	Yes	14 (3.45%)	6 (2.96%)		
VBAC	No	403 (99.26%)	201 (99.01%)	1	C
	Yes	3 (0.74%)	2 (0.99%)		
Entonox (N_2_O + O_2_)	No	246 (60.59%)	44 (21.67%)	<0.001	C
	Yes	160 (39.41%)	159 (78.33%)		
Oxytocin	No	203 (50.00%)	92 (45.32%)	0.316	C
	Yes	203 (50.00%)	111 (54.68%)		
Water immersion	No	226 (55.67%)	93 (45.81%)	0.027	C
	Yes	180 (44.33%)	110 (54.19%)		
Duration 1st stage (minutes)		222.71 (109.91 SD)	245.33 (123.54 SD)	0.036	W
Duration 2nd stage (minutes)		35.52 (28.91 SD)	37.64 (29.41 SD)	0.260	W
Delivery at night		95 (23.40 SD)	50 (24.63 SD)	0.814	C
Delivery during the day		311 (76.60 SD)	153 (75.37 SD)		
Episiotomy	No	166 (40.89%)	94 (46.31%)	0.235	C
	Yes	240 (59.11%)	109 (53.69%)		
Perineal tearing (I–IV)	No	329 (81.03%)	151 (74.38%)	0.083	C
	Yes	77 (18.97%)	52 (25.62%)		
Intact perineum	No	318 (78.33%)	160 (78.82%)	0.972	C
	Yes	88 (21.67%)	43 (21.18%)		
Birth weight (grams)		3380.15 (369.64 SD)	3393.45 (456.78 SD)	0.699	T
Umbilical cord blood pH		7.35 (0.07 SD)	7.36 (0.07 SD)	0.697	W
Umbilical cord blood pH < 7.2	No	392 (96.55%)	201 (99.01%)	0.128	C
	Yes	14 (3.45%)	2 (0.99%)		

* T—Student’s *t*-test, W—Wilcoxon test, C—chi-square test.

**Table 3:** Multivariate regression model—the effect of the variables on duration of the first stage of labour.

Variable	Estimate	SD	T	*p*	95% CI
Primiparity	89.460	8.720	10.259	<0.001	72.334
Water immersion	8.708	10.552	0.825	0.410	−12.015
TENS	−3.996	13.574	−0.294	0.769	−30.653
Entonox (N_2_O + O_2_)	−0.483	9.225	−0.052	0.958	−18.599
Water immersion–TENS	49.116	18.121	2.710	0.007	13.528

## Data Availability

The raw data supporting the conclusions of this article will be made available by the authors on request, as they contain sensitive personal information.
